# A Score-Based Approach to ^18^F-FDG PET Images as a Tool to Describe Metabolic Predictors of Myocardial Doxorubicin Susceptibility

**DOI:** 10.3390/diagnostics7040057

**Published:** 2017-10-26

**Authors:** Matteo Bauckneht, Silvia Morbelli, Francesco Fiz, Giulia Ferrarazzo, Roberta Piva, Alberto Nieri, Matteo Sarocchi, Paolo Spallarossa, Maria Elisa Canepari, Eleonora Arboscello, Andrea Bellodi, Massimo Massaia, Andrea Gallamini, Paolo Bruzzi, Cecilia Marini, Gianmario Sambuceti

**Affiliations:** 1 Nuclear Medicine, Policlinico San Martino Hospital and Department of Health Sciences, University of Genoa, 16132 Genoa, Italy; silviadaniela.morbelli@hsanmartino.it (S.M.); francesco.fiz.nm@gmail.com (F.F.); giulia.ferrarazzo@gmail.com (G.F.); pivaroberta@hotmail.it (R.P.); albertonieri@yahoo.it (A.N.); sambuceti@unige.it (G.S.); 2 Nuclear Medicine Unit, Department of Radiology, Uni-Klinikum, 72070 Tübingen, Germany; 3 Clinic of Cardiovascular Diseases, Policlinico San Martino Hospital, 16132 Genoa, Italy; sarocchi@gmail.it (M.S.); spallarossa@unige.it (P.S.); 4 Ematology Unit, ASO Santa Croce e Carle, 12100 Cuneo, Italy; me.canepari@libero.it (M.E.C.); massimo.massaia@unito.it (M.M.); 5 Clinic of Internal Medicine 3, Department Internal Medicine, Policlinico San Martino Hospital, 16132 Genoa, Italy; eleonora.arboscello@hsanmartino.it (E.A.); andreabellodi@libero.it (A.B.); 6 Department of Research, Innovation and Statistics, Antoine Lacassagne Cancer Centre, 06189 Nice, France; andreagallamini@gmail.com; 7 Epidemiology Unit, Policlinic San Martino Hospital, 16132 Genoa, Italy; paolo.bruzzi@hsanmartino.it; 8 CNR Institute of Bioimaging and Molecular Physiology, Section of Genoa, 20090 Milan, Italy; cecilia.marini@unige.it

**Keywords:** cardiotoxicity, doxorubicin, positron emission tomography, fluorodeoxy-glucose, myocardial metabolism, deauville score

## Abstract

Purpose: To verify the capability of ^18^F-fluorodeoxy-glucose positron emission tomography/computed tomography (FDG-PET/CT) to identify patients at higher risk of developing doxorubicin (DXR)-induced cardiotoxicity, using a score-based image approach. Methods: 36 patients underwent FDG-PET/CT. These patients had shown full remission after DXR-based chemotherapy for Hodgkin’s disease (DXR dose: 40–50 mg/m^2^ per cycle), and were retrospectively enrolled. Inclusion criteria implied the presence of both pre- and post-chemotherapy clinical evaluation encompassing electrocardiogram (ECG) and echocardiography. Myocardial metabolism at pre-therapy PET was evaluated according to both standardized uptake value (SUV)- and score-based approaches. The capability of the score-based image assessment to predict the occurrence of cardiac toxicity with respect to SUV measurement was then evaluated. Results: In contrast to the SUV-based approach, the five-point scale method does not linearly stratify the risk of the subsequent development of cardiotoxicity. However, converting the five-points scale to a dichotomic evaluation (low vs. high myocardial metabolism), FDG-PET/CT showed high diagnostic accuracy in the prediction of cardiac toxicity (specificity = 100% and sensitivity = 83.3%). In patients showing high myocardial uptake at baseline, in which the score-based method is not able to definitively exclude the occurrence of cardiac toxicity, myocardial SUV mean quantification is able to further stratify the risk between low and intermediate risk classes. Conclusions: the score-based approach to FDG-PET/CT images is a feasible method for predicting DXR-induced cardiotoxicity. This method might improve the inter-reader and inter-scanner variability, thus allowing the evaluation of FDG-PET/CT images in a multicentral setting.

## 1. Introduction

The use of systemic chemotherapy is the main form of treatment in case of hematological malignancies. While allowing a curative effect, its greatest drawback resides in the need to find a balance between therapeutic effectiveness and the onset of future complications, especially in the setting of younger patients. In fact, starting 30 years after treatment, the cumulative mortality from therapy-related medical illness actually exceeds that from cancer recurrence [1]. This phenomenon is attributable to both secondary malignancies and late cardiotoxicity [2,3,4] and can considerably impair life expectancy in younger patients. Moreover, the occurrence of cardiac injury can decisively affect both overall performance status as well as quality of life, particularly in elderly subjects, in patients with cardiovascular comorbidities, or in those in which additional thoracic irradiation is indicated [4,5,6,7]. In these regards, dose-dependent anthracycline-induced cardiomyopathy is one of the most acknowledged and well-studied clinical models of cardiovascular toxicity by chemotherapy.

The pathogenic mechanisms of late-onset anthracyclines-related myocardial damage has been to this day not fully explained. Several studies pointed out that doxorubicin (DXR), one of the most utilized anthracyclines, can specifically affect myocardial metabolic processes. Evaluation of in vivo glucose metabolism by means of molecular imaging confirmed this hypothesis, highlighting altered glucose uptake pattern, both in experimental animals and humans [8,9,10,11]. Accordingly, an in vitro study highlighted mitochondrial dysfunction in cardiomyocites exposed to DXR; this finding was associated with increased glucose consumption, possibly due to enhanced anaerobic glycolysis [12].

This pattern may be detected by ^18^F-fluorodeoxy-glucose positron emission tomography/computed tomography (FDG-PET/CT): increasing glucose consumption might in fact signal ongoing mitochondrial failure and a subsequent increase of the less energy-efficient glycolysis [12,13,14]. Similarly, low FDG-uptake at baseline might indicate a stricter dependence on mitochondrial-based ATP-generating processes and, thus, a greater susceptibility to DXR-linked damage [8].

For these reasons, the evaluation of FDG-PET/CT in patients treated with a DXR-based regimen might offer a viable window on metabolic changes within the myocardium, possibly identifying ongoing myocardial damage.

However, a major limitation is that glucose metabolism activity is evaluated by means of a standardized uptake value (SUV)-based approach, which might be influenced by several factors not related to tissue characteristics, including plasma glucose concentration, length of uptake period, partial volume effects, and recovery coefficient, as well as FDG-PET/CT scanner sensitivity [15]. The error margin due to these factors can actually exceed 50% [15].

This issue has been tackled in clinical nuclear medicine through the introduction of scales able to standardize metabolic qualitative estimation. An emblematic example is the five-point scale criteria (also called Deauville criteria), initially proposed for the evaluation of FDG-PET/CT of patients affected by Hodgkin’s disease (HD) [16]. This approach is based on the visual comparison of relative metabolic activity in the evaluated tissue with a reference area (mediastinal blood pool and liver) and showed an excellent inter-observer reproducibility [16]. Following success in HD, the five-point scale has been tentatively proposed for other malignancies, including non-Hodgkin’s lymphoma [17] and head and neck cancer [18], as well as for inflammatory diseases such as vasculitis [19,20].

Based on these considerations, the present study aimed: (1) to identify the capability of baseline FDG to identify patients at higher risk of developing DXR-induced cardiotoxicity using a five-point scale approach; and (2) to evaluate whether a score-based image evaluation is superior in predicting myocardial dysfunction with respect to the SUV-based approach in a retrospectively enrolled HD patient population, submitted to serial FDG-PET/CT before, during, and after DXR administration. To achieve this aim, the long-term correlation between the SUV- and score-based analysis of glucose analogue uptake and longitudinal myocardial function assessed by means of a follow-up clinical evaluation, ECG and transthoracic echocardiography were evaluated.

## 2. Materials and Methods

### 2.1. Patient Enrollment

The present study is a retrospective re-evaluation of FDG-PET/CT scans from a previously enrolled patient population [8]. The report of that study includes a through description of the materials and method. Briefly, the study population included HD patients evaluated by means of FDG-PET/CT performed in our institution from January 2008 to December 2015 according to the following population inclusion criteria: (1) no history of cardiovascular disease; (2) no diabetes; (3) availability of a clinical pre- and post-chemotherapy clinical evaluation encompassing electrocardiogram (ECG) and transthoracic echocardiography; (4) normal findings at pre-therapy clinical evaluation; (5) planned chemotherapy scheme (Adriamycin, Bleomycin, Vinblastine and Dacarbazine -ABVD-; DXR dose: 40–50 mg/m^2^ per cycle); (6) available staging FDG-PET/CT scan (PET1); (7) negative interim, post-therapy and 6 month follow up PET scan according to the Deauville criteria [17]; and (8) no subsequent HD relapse at late clinical follow-up. This selection process narrowed a final population of 36 patients.

### 2.2. FDG-PET/CT Scans

In accordance to our standard clinical PET protocol, FDG-PET/CT scans were performed using a 16-slice Biograph 16 PET/CT hybrid system (Siemens Medical Solutions). After a 6-h fasting state, each patient received an intravenous bolus of FDG (4.8–5.2 MBq/kg of body weight). PET/CT acquisition started 60–75 min afterward; in the meantime, the patients were hydrated and encouraged to urinate, so as to decrease the unbound tracer fraction. The entire body was scanned from vertex to mid-thigh in arms-up position. The emission scan lasted 120 s per bed position. PET raw data were reconstructed using ordered-subset expectation maximization (3 iterations and 16 subsets), and attenuation was corrected using the raw CT data. Then, a 16-detector-row helical CT was performed with non-diagnostic current and voltage settings (120 kV and 80 mA), a gantry rotation speed of 0.5 s, and a table speed of 24 mm per gantry rotation. No contrast medium was injected. The entire CT dataset was fused with the 3-dimensional PET images using an integrated software interface (Syngo; Siemens Healthcare Headquarters, Erlangen, Germany).

### 2.3. Image Analysis

**Figure 1:**
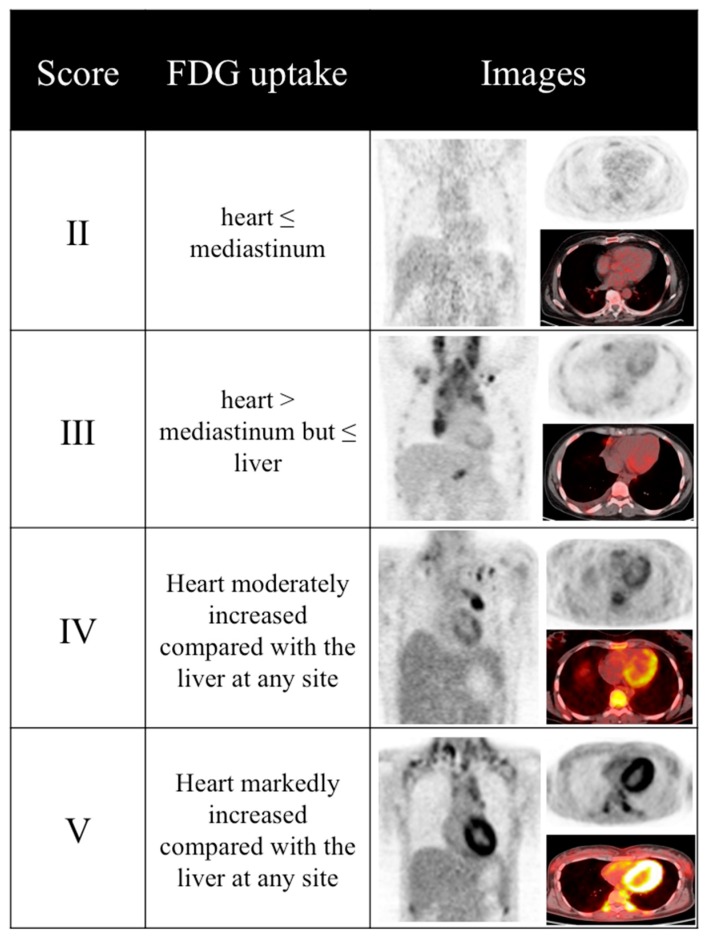
Score-based approach to ^18^F-fluorodeoxy-glucose positron emission tomography/computed tomography (FDG-PET/CT) images. Examples of score-based evaluation of myocardial FDG uptake among the 36 enrolled Hodgkin’s disease (HD) patients. None of PET1 myocardial images was classified as score 1.

Patients’ data were anonymized before executing the retrospective analysis. Only PET1 images were evaluated. The SUV-based image analysis was performed as detailed elsewhere [8]. Briefly, an experienced nuclear medicine physician, unaware of cardiotoxic patient’s profile, manually drew a volume of interest (VOI) on the metabolically active left ventricular myocardium to estimate SUVmean and SUVmax (termed LV-SUVmean and LV-SUVmax, respectively). Subsequently, the same images were visually evaluated focusing on myocardium, following five-point scale criteria [16]. In case of heterogeneous myocardial FDG uptake, the highest uptake was scored and compared to reference tissues. In patients showing mediastinum or liver disease, only healthy tissue uptake was considered as reference. Examples of corresponding PET images to different score classes are shown in Figure 1.

### 2.4. Clinical Data Retrospective Analysis

In accordance to the standard clinical protocol of our institution, pre- and post-chemotherapy cardiac evaluations were performed, encompassing symptoms-focused anamnesis (palpitations, syncope, chest pain or dyspnea), physical examination, and baseline ECG. A transthoracic echocardiogram was also performed to estimate wall thickness, left ventricular (LV) diameters and ejection fraction (EF) calculated with Simpson method [21]. LV diastolic function was assessed by the E/A wave ratio and by E wave deceleration time [21].

### 2.5. Statistical Analysis

All data are presented as mean ± standard deviation (SD). Differences between paired and unpaired data were analyzed through Student *t*-test and the chi-squared test for categorical variables. The capability of the score-based image investigation to predict cardiotoxicity was tested by means of a univariate analysis.

Furthermore, receiver-operating characteristic (ROC) curve analysis was performed to evaluate the capability of both five-point scale and LV-SUV to discriminate between individuals who developed or not cardiac toxicity and to determine the corresponding cut-off values. As post hoc analysis, this value was tested to identify the best predictor of cardiotoxicity. A probability value *p* < 0.05 was considered statistically significant. A biomedical statistician performed the statistical analyses using a dedicated software application (SPSS, IBM Corp. Released 2012, Version 21.0. IBM Corp.: Armonk, NY, USA).

## 3. Results

### 3.1. Clinical Data and Myocardial Functional Outcome

Demographic and clinical data of patient population are detailed elsewhere [8]. Median follow-up time between the end of chemotherapy and clinical re-evaluation was 27 months (range: 8–96 months). None of the 36 enrolled patients reported any hospitalization potentially related to cardiac disorders. According to the inclusion criteria, pre-therapy LV dimensions and function were remarkably similar in all patients. However, in the later clinical follow-up, ECG or echocardiography documented new onset abnormalities in 11/36 patients (30%, 4 females, mean age 44 ± 17, age range 21–66). Appearance of cardiac abnormality was not related to differences in age, gender, body weight, gycaemia at tracer injection and cardiovascular risk profile (including hypertension, tobacco use, total cholesterol, LDL, triglycerides, creatinine and family history of coronary artery disease), mediastinal radiotherapy, total DXR dose, or follow-up duration [8]. According to the population inclusion criteria none of the 36 patients was affected by diabetes.

### 3.2. Score-Based vs. SUV-Based Image Analysis

As previously showed, a baseline SUV-based image analysis is able to predict DXR-induced cardiac toxicity. Results of SUV-based analysis of images are detailed elsewhere [8]. Briefly, the 11 “cardiotoxic” patients displayed markedly lower myocardial SUV mean values at baseline with respect to the remaining 25.

Details about the relationship between the occurrence of DXR-related cardiac toxicity and score-based images evaluation are summarized in Table 1. The five-point scale does not linearly stratify the risk of the subsequent development of cardiac toxicity. Based on this finding, a post hoc analysis was conducted, converting the 5-point scale to a dichotomic evaluation. A score of <3 was classified as “low myocardial uptake”, while a score ≥3 was termed as “high myocardial uptake”. This latter approach showed a high accuracy (specificity = 100% and sensitivity = 83.3%, Table 2).

Subsequently, the cut-off SUV-based and dichotomist score-based approaches were compared (Table 3). In patients showing a baseline high myocardial uptake, the LV-SUVmean is able to further stratify the risk between low (LV-SUVmean > 2.015) and intermediate (LV-SUVmean < 2.015) risk classes.

## 4. Discussion

In keeping with our previous findings [8], showing that low myocardial FDG uptake is able to early identify patients at higher risk of developing DXR-induced cardiac toxicity, in the present study we demonstrated that not only the SUV-based but also a score-based approach to FDG-PET/CT images can be proposed in the clinical setting. Accordingly, both these indicators showed a good capability to predict the subsequent development of cardiac toxicity. However, the score-based evaluation of images allowed the same accuracy with easier standardization. This approach might, therefore, improve the inter-reader and inter-scanner variability, thus allowing the evaluation of FDG-PET/CT images as a tool to predict DXR cardiotoxicity in a multicentric setting.

SUV is the most largely used indicator of glucose analogue avidity in PET cancer studies. This value is defined as the tissue concentration of tracer as measured by a PET scanner divided by the ratio between injected activity and body weight [22]. This index accounts for both the time elapsed from tracer injection and corrects for the distribution. However, numerous patient- and equipment-linked factors cause significant discrepancies between scans [15]. Consequently, the cut-off value obtained by our analysis might not be valid in independent patient populations. On the other hand, SUV did not add any added predictive value compared with visual inspection in the clinical setting [23,24]. Differently from SUV-based image analysis, the score-based approach is less affected by confounding variables known to impact on SUV measurement. Accordingly, the score is chosen by means of a qualitative interpretation on focal uptake in relation to uptake in adjacent tissues, which are influenced by technical factors by the same degree, with respect to the target tissue. Therefore, this method is able to reduce inter-institutional variations.

Moreover, in our patient population, low baseline myocardial uptake, defined as five-point scale score of <3, showed a high capability in prediction of cardiac toxicity, with a positive predictive value of 100%. In contrast, the opposite finding (high myocardial uptake, define as five-point scale score ≥3) could not discriminate between high- and low-risk patients. As shown by our post hoc analysis, in this subset of patients (high myocardial uptake) the cut-off value of LV-SUVmean = 2.015 is able to distinguish between low (LV-SUVmean < 2.015, in which the percentage of cardiotoxic patients was 0%) and intermediate risk patients (LV-SUVmean < 2.015, in which the percentuage of cardiotoxic patients was 31%). However, for the reasons explained above, this cut-off value, which is data-driven, can be considered valid only in the present patient population, being theoretically dependent by technical factors.

## 5. Limitations

This study presents some limitations. Firstly, due to the retrospective nature of the study, the size of the study is limited to 36 patients. As a matter of fact, it depends by our strict population inclusion criteria. However, these criteria allowed the identification of subjects in which the ABVD scheme was administered with standard doses. On the other hand, due to the retrospective nature of the study, a specific high-fat low-carbohydrate diet or pharmacologic preparation was not administered prior to the FDG-PET/CT scan. Consequently, the analysis of DXR effect on myocardial FDG uptake might have been hampered by the large variability of myocardial metabolic pattern under fasting conditions, which is at least partially related to dietary regimen in the days before the scan. As a consequence, our findings need to be confirmed by larger, multicentral, prospective studies.

## 6. Conclusions

The score-based approach to FDG-PET/CT images is a feasible method for predicting cardiac toxicity in a population of young patients treated with DXR. With respect to SUV-based approach, this method might, therefore, improve the inter-reader and inter-scanner variability, allowing the evaluation of FDG-PET/CT images as a tool to predict DXR cardiotoxicity in a multicentral setting.

## 7. Compliance with Ethical Standards

All procedures performed in the present study were in accordance with the 1964 Helsinki declaration and its later amendments or comparable ethical standards. The institutional review board approved this study and all subjects signed a written informed consent related to the imaging procedure, as part of our routine clinical care.

## Figures and Tables

**Table 1:** Relationship between DXR-related damage and score-based images evaluation.

			Myocardial Damage		Total
			0	1.0	
Score 2–5	2	Count	0	6	6
		% in Score 2–5	0.0%	100.0%	100.0%
	3	Count	9	1	10
		% in Score 2–5	90.0%	10.0%	100.0%
	4	Count	5	3	8
		% in Score 2–5	62.5%	37.5%	100.0%
	5	Count	11	1	12
		% in Score 2–5	91.7%	8.3%	100.0%
Total		Count	25	11	36
		% in Score 2–5	69.4%	30.6%	100.0%

**Table 2:** Relationship between DXR-related damage and binary images evaluation.

			Myocardial Damage		Total
			0	1.0	
ScoreBin	score: <3	Count	0	6	6
		% in ScoreBin	0.0%	100.0%	100.0%
	Score: ≥3	Count	25	5	30
		% in ScoreBin	83.3%	16.7%	100.0%
Total		Count	25	11	36
		% in ScoreBin	69.4%	30.6%	100.0%

**Table 3:** Comparison between cut-off SUV-based and dichotomist score-based evaluation.

Score				Myocardial Damage		Total
				0	1	
Score < 3	Binary SUV myocardium	<2	Count		6	6
			% in binary SUV myocardium		100.0%	100.0%
	Total		Count		6	6
			% in binary SUV myocardium		100.0%	100.0%
Score ≥ 3	Binary SUV myocardium	Normal	Count	14	0	14
			% in binary SUV myocardium	100.0%	0.0%	100.0%
		<2	Count	11	5	16
			% in binary SUV myocardium	68.8%	31.3%	100.0%
	Total		Count	25	5	30
			% in binary SUV myocardium	83.3%	16.7%	100.0%
Total	Binary SUV myocardium	Normal	Count	14	0	14
			% in binary SUV myocardium	100.0%	0.0%	100.0%
		<2	Count	11	11	22
			% in binary SUV myocardium	50.0%	50.0%	100.0%
	Total		Count	25	11	36
			% in binary SUV myocardium	69.4%	30.6%	100.0%
